# Antibiofilm, Antimicrobial, Anti-Quorum Sensing, and Antioxidant Activities of Saudi Sidr Honey: In Vitro and Molecular Docking Studies

**DOI:** 10.3390/pharmaceutics15092177

**Published:** 2023-08-22

**Authors:** Abdulrahman S. Bazaid, Ahmed Alsolami, Mitesh Patel, Aiah Mustafa Khateb, Abdu Aldarhami, Mejdi Snoussi, Shekah M. Almusheet, Husam Qanash

**Affiliations:** 1 Department of Medical Laboratory Science, College of Applied Medical Sciences, University of Ha’il, Hail 55476, Saudi Arabia; h.qanash@uoh.edu.sa; 2 Department of Internal Medicine, College of Medicine, University of Ha’il, Hail 55476, Saudi Arabia; a.alsolami@uoh.edu.sa; 3 Department of Biotechnology, Parul Institute of Applied Sciences and Centre of Research for Development, Parul University, Vadodara 391760, Gujarat, India; patelmeet15@gmail.com; 4 Medical Laboratory Technology Department, College of Applied Medical Sciences, Taibah University, Madinah 42353, Saudi Arabia; akhateb@taibahu.edu.sa; 5 Special Infectious Agents Unit-BSL3, King Fahd Medical Research Center, King Abdulaziz University, Jeddah 21362, Saudi Arabia; 6 Department of Medical Microbiology, Qunfudah Faculty of Medicine, Umm Al-Qura University, Al-Qunfudah 21961, Saudi Arabia; ahdarhami@uqu.edu.sa; 7 Department of Biology, College of Science, University of Ha’il, Hail 55476, Saudi Arabia; snmejdi@yahoo.fr; 8 Laboratory of Genetics, Biodiversity and Valorization of Bio-Resources, University of Monastir, Higher Institute of Biotechnology of Monastir, Avenue Tahar Haddad, BP74, Monastir 5000, Tunisia; 9 Department of Laboratory, King Khalid Hospital, Hail 55421, Saudi Arabia; shekhahmubarak@gmail.com

**Keywords:** Saudi Sidr honey, antimicrobial, antioxidant, anti-quorum, ADMET

## Abstract

Sidr honey is a valuable source of bioactive compounds with promising biological properties. In the present study, antimicrobial, antioxidant, and anti-quorum sensing properties of Saudi Sidr honey were assessed, along with phytochemical analysis, via gas chromatography–mass spectrometry (GC-MS). In silico study was also carried out to study the drug-likeness properties of the identified compounds and to study their affinity with known target proteins assessed using molecular docking approach. The results showed that Saudi Sidr honey exhibited promising antibacterial activity, with MIC values ranging from 50 to 400 mg/mL and MBC values from 50 to >450 mg/mL. Interestingly, the Saudi Sidr honey was active against *Candida auris* and *Candida neoformans*, with an MIC value of about 500 mg/mL. Moreover, the Sidr honey showed important antioxidant activities (ABTS assay: IC50 5.41 ± 0.045 mg/mL; DPPH assay: IC50 7.70 ± 0.065 mg/mL) and *β*-carotene bleaching test results (IC50 ≥ 20 mg/mL). In addition, the Saudi Sidr honey was able to inhibit biofilm formation on glass slides at 1/2 MIC by 77.11% for *Bacillus subtilis*, 70.88% for *Staphylococcus aureus*, 61.79% for *Escherichia coli*, and 56.64% for *Pseudomonas aeruginosa*. Similarly, violacein production by *Chromobacterium violaceum* was reduced by about 56.63%, while the production of pyocyanin by *P. aeruginosa* was decreased to 46.27% at a low concentration of Saudi Sidr honey. ADMET properties showed that five identified compounds, namely, 1-cyclohexylimidazolidin-2-one, 3-Butyl-3-methylcyclohexanone, 4-butyl-3-methoxy-2-cyclo penten-1-one, 2,2,3,3-Tetramethyl cyclopropane carboxylic acid, and 3,5-dihydroxy-2-(3-methylbut-2-en-1-yl showed promising drug-likeness properties. The compound 3,5-dihydroxy-2-(3-methylbut-2-en-1-yl exhibited the highest binding energy against antimicrobial and antioxidant target proteins (1JIJ, 2VAM, 6B8A, 6F86, 2CDU, and 1OG5). Overall, the obtained results highlighted the promising potential of Saudi Sidr honey as a rich source of bioactive compounds that can be used as food preservatives and antimicrobial, antioxidant, and anti-quorum sensing molecules.

## 1. Introduction

Traditionally, honey is a natural product that is derived from the nectar of flowers, which is collected by honeybees [1]. It is estimated that humans have been using honey since ancient times, roughly 5500 years ago [2]. There has been a long history of people in the ancient world consuming honey for its nutritional benefits, as well as its medicinal benefits, including Greeks, Chinese, Egyptians, Romans, Mayans, and Babylonians [2,3,4,5]. Unlike other natural products, honey is the only insect-derived product that is nutritionally, cosmetically, therapeutically, and industrially valuable [6,7,8,9]. It has been proven to be a beneficial addition to a balanced diet for both females and males of all ages [10,11]. At the present time, as evidenced by several studies, it appears to be capable of exerting a number of health-beneficial effects, such as antioxidant, anti-inflammatory, antibacterial, antidiabetic, respiratory, gastrointestinal, cardiovascular, and nervous system protective effects [12].

The composition of honey contains a wide variety of constituents in small quantities, including minerals, free amino acids, proteins, vitamins, enzymes, organic acids, flavonoids, phenolic acids, and various other organic acids, as well as a variety of phytochemical compounds [13,14]. The number of these components contained in honey depends on a variety of factors, such as the geographical origin of honey, the source of the flowers, the weather conditions, and any treatments that were applied [15,16]. Processing, handling, and storing honey can affect its composition in a variety of ways [17,18]. There is also a correlation between the quality of honey and the number of floral resources, as well as the treatment by beekeepers [19].

Although different kinds of honey have similar physico-chemical properties, no two honeys are the same, unless they are harvested from the same hive at the same time, demonstrating the differences between types of honey in their composition and properties. Accordingly, honey has been extensively examined in terms of its botanical origin, geography, and physico-chemical properties to assess whether there is a significant variation in these factors among different honey types [17,20,21,22,23,24,25]. Several factors, such as the harvesting season and associated weather conditions, as well as processing techniques used after harvest, can also affect its variations [26].

Sidr honey is derived from the nectar of the *Ziziphus* or Lote tree (*Ziziphus spina-christi*, *Ziziphus lotus*, or *Ziziphus jujuba*). In the Middle East, this particular tree is commonly found in the desert regions of countries such as Yemen and Saudi Arabia, as well as in Pakistani territory in the Potohar region. However, this tree is primarily found in Yemen, where many people use all parts of the tree for medicinal purposes [20]. Due to the fact that the entire tree is believed to possess healing properties, it is obvious that the same also applies to the nectar from the tree, which means that honey can be used to treat any kind of sickness or illness. In addition to being used in cooking a variety of foods and sweets, Sidr honey is also used as a smoothening agent for hair, as well as in a variety of cosmetics and ayurvedic products [27,28].

Sidr honey is used to treat a wide variety of diseases across the globe, including liver disease, stomach ulcers, lung infections, malnutrition, digestion difficulties, constipation, eye infections, wound infections, and general health conditions. Bacteria can form a bacterial community called a biofilm which is resistant to antibiotics and thus considered a challenging global health problem [29]. Honey has shown activity to inhibit biofilm formation in pathogenic bacteria including *Staphylococcus aureus* [29,30]. Therefore, the present study was conducted with the aim of conducting phytochemical analyses and to further investigate the antimicrobial, antioxidant, anti-quorum sensing, and antibiofilm properties of Saudi Sidr honey to evaluate the potential uses of the tested honey in more detail.

## 2. Materials and Methods

### 2.1. Collection of Honey Sample 

A sample of Sidr honey which was purchased from a local market of Saudi Arabia (Al-Baha city) in January 2022 was immediately transferred to the laboratory and began being processed. It was found that the honey was free of any signs of granulation, fermentation, or contamination. For the purpose of conducting further studies, the honey was maintained at a normal laboratory temperature.

### 2.2. GC-MS Analysis of the Saudi Sidr Honey

To determine the composition of the Saudi Sidr honey via gas chromatography–mass spectrometry (GC-MS) analysis, a Shimadzu Nexis GC-2030 and QP2020 NX MS were utilized. An SH-Rxi-5Sil (30 m, 0.25 mm ID, 0.25 µm df, Shimadzu, Kyoto, Japan) column was used to separate the sample. After adjusting the temperature to 50 °C for 3 min, a rate of 5 °C per minute was applied until 250 °C was reached and, after a further 10 min, the temperature was raised to 270 °C for final separation. The system was filled with a total of 20 µL of sample and helium was used as a carrier gas for carrying the sample. The peaks obtained from the GC-MS separation were compared to the NIST database to determine the probable composition of the honey [31]. 

### 2.3. Screening of Antibacterial Activity

To conduct in vitro antibacterial activity of Saudi Sidr honey, a series of clinical isolates of bacterial strains, such as *Staphylococcus aureus*, *Pseudomonas aeruginosa*, *Escherichia coli*, and MDR *Acinetobacter baumannii* were obtained from the King Khalid general hospital in Hail, Saudi Arabia. Several bacterial strains, such as *Bacillus subtilis* (MTCC 121), *S. aureus* (MTCC 96), *E. coli* (MTCC 9537), and *P. aeruginosa* (MTCC 741), were also obtained from the Microbial Type Culture Collection (MTCC), Chandigarh, India. A Muller-Hinton Agar (MHA) (HiMedia^®^, Mumbai, India) plate was used to maintain the bacterial strains that were obtained [32].

#### 2.3.1. Determination of Minimum Inhibitory Concentration (MIC) against Bacterial Isolates

The MIC of Saudi Sidr honey against different bacterial pathogens was determined using the broth dilution method [33]. Inoculums were prepared from respective bacteria suspensions that had been cultured overnight in MHB (HiMedia^®^, Mumbai, India). In sterile 96-well plates, the honey sample was diluted with sterile distilled water and different concentrations (10–500 mg/mL) were added aseptically into the wells (100 µL per well). To each of the corresponding wells, cultures of each bacterium (10^8^ CFU/mL) were added and incubated at 37 °C for 24 h. Upon completion of the incubation period, absorbance was read at 620 nm using a multimode microtiter plate reader (BioTek, Winooski, VT, USA). Based on the results of the test, the MIC value was determined as the lowest concentration of the drug that visually inhibits bacterial growth. During the experiment, two controls were used: a negative control consisted of MHB containing no bacteria at all, while a positive control consisted of bacterial suspension containing no honey sample (untreated). A triplicate experiment was carried out to obtain MIC values and the results are expressed in terms of mg/mL.

#### 2.3.2. Fungi Minimum Inhibitory Concentration (MIC) Evaluation 

To evaluate the antifungal effectiveness of Saudi Sidr honey, the measurement of the MIC was made using the broth microdilution technique. The CLSI protocol (NCCLS-M27-A, 2002) was adapted for usage. To make the stock solution, 3 g of honey was briefly diluted in 1.5 mL of sterile water. To speed up dissolving, the liquid was heated at 50 °C for 15 min. In sterile water, a series of dilutions of honeys (2000, 1800, 1600, 1400, 1200, and 1000 mg/mL) were made. All concentration was aliquoted into a sterile, 96-well round-bottom plate at a volume of 100 µL. A quantity of 100 µL of a fungus suspension made in Sabouraud dextrose broth (HiMedia^®^, Mumbai, India) (optical density of 0.08) was pipetted into each well and stirred. Each plate included two wells of positive controls (fungal suspension) and three wells of negative controls (broth on its own, sterile water, and diluted honey). After incubating for 24 h at 30 °C, growth was observed for visual turbidity. The MIC was defined as the lowest amount of honey that could not allow the test microorganisms to proliferate. Each MIC value is provided in µg/mL and three replicas of each experiment were performed.

#### 2.3.3. Determination of Minimum Bactericidal Concentration (MBC)

To determine the values of MBC following the MIC assay, 5 µL of the samples were spread on MHA plates (HiMedia^®^, Mumbai, India) from the wells where no signs of bacterial growth were evident. In the following step, the plates were incubated at 37 °C for an incubation period of 18 to 24 h. The MBC concentration was recorded at the end of the incubation period that yielded three or fewer colonies, meaning that 99% of the inoculum had been killed at the lowest concentration [34]. The MBC/MIC ratio was calculated in response to the fact that antibacterial agents are bactericidal if the MBC value is no greater than four times the MIC value [35]. 

### 2.4. Antibiofilm Assay of Saudi Sidr Honey 

Sidr honey was evaluated for its ability to inhibit biofilms using a spectroscopic assay according to a previously published study [36]. To microtiter plates, bacterial suspensions (100 µL) and Sidr honey (1/2 MIC) were added, and the plates were incubated for 24 h at 37 °C. In the following steps, planktonic cells were removed, and wells were washed very delicately with PBS (200 µL) (Hi-Media, India). The biofilms formed by adherent bacterial cells were stained with 0.1% crystal violet stain (100 µL) (HiMedia^®^, Mumbai, India) and incubated at 37 °C for 30 min. The extra stain was washed off with PBS, and 200 µL of 95% ethanol was added to each stained well. The wells were then incubated at 37 °C for 15 min to aid in the solubility of the dye. The absorbance of each well was measured spectrophotometrically at 590 nm using a UV-Visible spectrophotometer (UV-1800, Shimadzu, Japan). To estimate the percentage of inhibition, the following equation was used:

[OD (control) − OD (test)/OD (control)] × 100.

### 2.5. Determination of Antibiofilm Activity by Light Microscopy (LM)

The biofilms formed by bacterial strains on glass coverslips were visualized according to the method previously described [37]. The bacterial cultures (500 μL) were inoculated into 6-well plates containing a coverslip (1 × 1 cm) in MHB supplemented with 0.2% glucose (HiMedia^®^, Mumbai, India). For treatment, 500 μL of Sidr honey (1/2 MIC) was added to the same well. Biofilms formed on glass coverslips after 48 h of incubation at 37 °C were gently detached and washed with PBS. Using the 0.1% crystal violet staining solution (HiMedia^®^, Mumbai, India), the biofilm was stained, following which it was examined under a light microscope with a magnification of 40× (Axioscope A1, Zeiss, Jena, Germany).

### 2.6. Inhibition of Quorum Sensing (QS) by Saudi Sidr Honey 

The Saudi Sidr honey was evaluated by means of a well diffusion assay for anti-QS activity against Chromobacterium violaceum (MTCC2656) as well as *P. aeruginosa* (MTCC2488). Overnight grown cultures (100 µL) of both bacterial strains were spread over the Luria Bertani agar (HiMedia^®^, Mumbai, India) plates and wells were made at the center of the plates with the help of a cork borer. Approximately 60 µL of Sidr honey sample was injected into each of the wells, and the plates were then incubated at 37 °C for 24 h to observe anti-QS effects. After 24 h, the inhibitory zones of tested bacteria were determined, and these inhibitory zones indicated that the treatment was anti-QS in nature [38]. By using the same method that was described above, MIC values were also determined.

### 2.7. Quantitative Analysis of Violacein Production in C. violaceum

A culture of *C. violaceum* was grown in LB medium in the absence and in the presence of Sidr honey (1/2 MIC) at 28 °C for 24 h. After incubation, culture was centrifuged at 10,000 rpm for 10 min to collect the cell pellet. The collected cell pellet was then dissolved in 1 mL DMSO (HiMedia^®^, Mumbai, India) and used for the quantification of violacein. The dissolved cell pellet was further centrifuged at 10,000 rpm for 10 min to remove cell debris and absorbance of supernatant was determined at 600 nm [39]. The determination of the inhibition in violacein production was estimated via following equation:

% Violacein inhibition = (ODcontrol − Odtest/ODcontrol) × 100

### 2.8. Quantitative Analysis of Pyocyanin Production in P. aeruginosa 

Using the method described by Essar et al. (2016) [40], pyocyanin pigment production from culture supernatants of *P. aeruginosa* was determined in the absence and presence of Sidr honey. The first step of the procedure was to extract 1.5 mL of culture supernatant of *P. aeruginosa*, either untreated or treated with a sub-MIC (1/2MIC), with 3 mL chloroform (HiMedia^®^, Mumbai, India), followed by re-extraction with 0.2 M HCl (700 µL) (HiMedia^®^, Mumbai, India) after the first extraction. As a next step, the solution was used for the measurement of the absorbance at 595 nm. The following formula was used to quantify pyocyanin production:

% Pyocyanin inhibition = (OD control − OD test/OD control) × 100

### 2.9. Evaluation of the Antioxidant Activities

Different assays were used to determine antioxidant activity of Saudi Sidr honey, such as 1,1-diphenyl-2-picrylhydrazyl (DPPH) radical scavenging activity, 2,2’-azino-bis(3-ethylbenzothiazoline-6-sulfonic acid) (ABTS) radical scavenging activity, and *β*-carotene bleaching assay, according to methods previously described [41].

#### 2.9.1. Scavenging Activity of DPPH Free Radicals

DPPH free radical scavenging activity was tested for Saudi Sidr honey based on the method previously described [31]. The DPPH solution (20 mg/L) (Merck KGaA, Darmstadt, Germany) was prepared by dissolving 2 mg of it in 100 mL of methanol. A solution of 1.5 mL of DPPH solution was added to different concentrations of methanolic honey, ranging from 20 to 40 mg/mL. After incubating the sample at 25 °C for 15 min, the absorbance of the sample was measured at 517 nm. During the study, the concentration of ascorbic acid was taken as a reference. To determine the scavenging ability of DPPH, the following formula was used. The concentration of honey required to scavenge 50% of DPPH∙ (IC_50_) was also calculated.

$$\mathrm{DPPH}\;\mathrm{scavenging}\;\mathrm{activity}\;(\%)=\frac{A_{control}-A_{sample}}{A_{control}}\times 100$$

A_control_ = absorbance of control.

A_sample_ = absorbance of sample.

#### 2.9.2. Scavenging Activity of ABTS Free Radicals 

In accordance with Re et al. [42], Sidr honey was evaluated for its ABTS free radical scavenging activity. As a first step, distilled water (dH_2_O) was used to prepare an ABTS solution (7 mM) (Merck KGaA, Darmstadt, Germany). Following the preparation of the ABTS, the ABTS was mixed with potassium persulfate (2.454 mM) to form ABTS (radical cation). Before using the generated ABTS, it was placed at room temperature in the dark for 12–16 h. The prepared ABTS was further diluted with dH_2_O until the absorbance was at a point of 0.70 (at 734 nm) (UV-1800, Shimadzu, Japan). Then, the honey sample (0.07 mL) and the ABTS (3 mL) were mixed together and incubated at room temperature for 7 min in the dark and the absorbance of the mixture was measured using a spectrophotometer at 734 nm. To calculate antioxidant activity, the following equation was used:

$$\%\;\mathrm{Inhibition}=\frac{A_{control}-A_{sample}}{A_{control}}\times 100$$

where A_control_ = absorbance of negative control at the moment of solution preparation and A_sample_ = absorbance of sample after incubation.

#### 2.9.3. *β*-Carotene Bleaching Assay

The *β*-carotene linoleate model system was examined as previously described [31]. Using a 100 mL round-bottom flask, 2 mL of *β*-carotene (0.2 g/L) (Merck KGaA, Darmstadt, Germany) was dissolved in chloroform followed by the addition of 0.02 mL of linoleic acid and 0.2 mL of Tween 20. Infusion of the mixture with 0.2 mL of Saudi Sidr honey solution was then carried out. Under vacuum at room temperature, after the evaporated material in the flask had reached a dry state, 50 mL of dH_2_O was added to it. Vigorous agitation of the mixture was carried out to form an emulsion. A total of 2 mL of the emulsion was transferred to another test tube and immediately placed in a water bath at 50 °C for 16 h. Over a period of 2 h, UV-Visible spectrophotometers were used to measure the absorbance of the sample every 20 min. As a standard, butylated hydroxytoluene (200 mg/mL) was used to construct the calibration curve. The results of the experiments are expressed as an average of three replicates. The following formula was used to calculate the bleaching activity of *β*-carotene (CBI), which is expressed as a percentage:

$$\mathrm{CBI}\;(\%)=\frac{B_{control}-B_{sample}}{B_{control}}\times 100$$

where B_control_ and B_sample_ represent the bleaching rates of *β*-carotene in the control and the sample, respectively.

### 2.10. ADMET Prediction

To filter out compounds from Saudi Sidr honey based on ADMET properties, the identified compounds were subjected to GC-MS analysis. A SwissADME, ADMETLAB 2.0, and Protox-II server for toxicity analysis was used for the prediction of ADME properties and PAINS (Pan-assay interference compounds) evaluation [43]. After selecting compounds with well-characterized ADMET properties, they were filtered for any PAINS patterns as well as the BBB (blood brain barrier) [44]. It has been shown that the PAINS filter can help avoid compounds having specific patterns that have a tendency to bind to multiple targets more often. The ADMET evaluation helps in the identification of compounds with drug-like properties in terms of their physico-chemical and pharmacokinetics characteristics, which reduces the chances of their failure in clinical trials [45].

### 2.11. Ligand and Protein Selection

To investigate the interaction between the identified components of Saudi Sidr honey with target proteins of antibacterial and antioxidant properties, molecular docking was performed. Crystal structures of antibacterial proteins, such as TyrRS of *S. aureus* (PDB—1JIJ.pdb), FtsZ of *B. subtilis* (PDB—2VAM.pdb), MvfR of *P. aeruginosa* (PDB—6B8A.pdb), GyraseB of *E. coli* (PDB—6F86.pdb) and crystal structures of antioxidant proteins, NAD(P)H oxidase (PDB—2CDU.pdb), and human cytochrome P450—1OG5 (PDB—1OG5.pdb) were retrieved from RCSB PDB. Three-dimensional structures of five compounds, which passed from ADMET properties such as 1-cyclohexylimidazolidin-2-one, 3-butyl-3-methylcyclohexanone, 4-butyl-3-methoxy-2-cyclopenten-1-one, 2,2,3,3-tetramethyl cyclopropane carboxylic acid, and 3,5-dihydroxy-2-(3-methylbut-2-en-1-yl) were retrieved from the well-known organic compound database PubChem in .sdf format. Using Open Babel, these compounds were converted to .pdb [46].

### 2.12. Molecular Docking Analysis

A three-dimensional structure of a protein derived from the RCSB PDB was prepared for molecular docking using AutoDock Tools (v1.5.7). AutoDock Tools provides various tools for preparing both the ligand and the target for docking, such as adding or removing hydrogen, repairing residues, and modifying protonation states. The protein structure was prepared via AutoDock MGL Tools using inbuilt AutoDock functions in which water molecules and ligands were removed and the structure was further repaired, after which polar hydrogen bond and Kollman charges were added and later saved in .pdbqt format. Structure files (.pdbqt-format) of six proteins and five compounds were docked separately using the molecular docking software AutoDock Vina (Version 1.5.7). All the parameters used for docking of five compounds with the receptors were kept the same. Auto Grid was used to prepare the grid map using a grid box [47]. The grid size was set to 43.897 × 67.024 × 51.535 xyz points for the 1JIJ receptor, 51.387 × 52.609 × 61.632 xyz points for 2VAM, 52.295 × 50.405 × 43.995 for 6B8A, 36.640 × 45.212 × 48.149 for 6F86, 64.419 × 62.664 × 68.619 for 2CDU, and 51.935 × 59.833 × 65.883 for 1OG5. Grid spacing was kept to 0.375 Å for all the receptors. The grid center for 1JIJ was designated at the following dimensions (x, y, and z): −11.673, 17.303, and 91.750; for 2VAM at (x, y, and z) 28.961, −8.988, and −1.985; for 6B8A at (x, y, and z) 27.692, −17.879, and 2.022; for 6F86 at (x, y, and z) 67.318, 32.048, and 54.475; for 2CDU at (x, y, and z) 10.187, 0.661, and 6.126; and for 1OG5 at (x, y, and z) −21.047, 78.257, and 30.100. For ligand translation and rotation to occur, a grid box was constructed in such a way that it encloses the entire binding site of both receptors while providing enough space for ligands to rotate and translate. The docked conformations were ranked by predicted binding energies, and the topmost docked conformation with the highest predicted binding energy was analyzed using BIOVIA Discovery Studio [48] to determine whether there is any intermolecular hydrogen bonding between active site amino acids of the receptors and docked ligands.

## 3. Results

### 3.1. GC-MS Analysis of Saudi Sidr Honey

**Figure 1:**
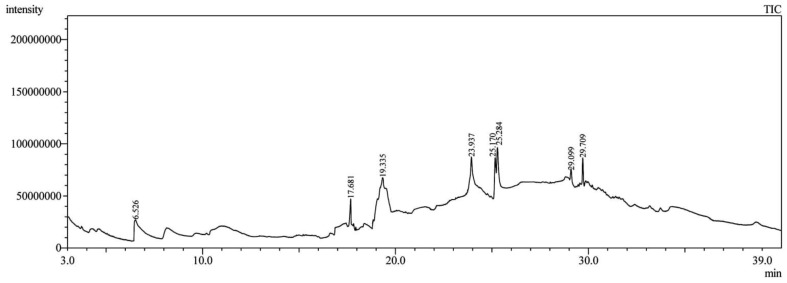
GC-MS chromatogram of Saudi Sidr honey.

An analysis of Saudi Sidr honey was carried out using GC-MS analysis to determine its chemical composition. Phytoconstituents were separated and identified using this technique based on their retention time, database difference (library), experimental *m*/*z*, MS fragments, metabolite class, and proposed compounds. As a result of the GC-MS analysis, different types of bioactive compounds were identified (Figure 1). The identified compounds are documented in Table 1.

### 3.2. Antibacterial Potential of Saudi Sidr Honey

The Saudi Sidr honey was examined qualitatively and quantitatively to determine its potential antagonistic activity against different clinical and reference bacterial strains. Based on the results of the antibacterial test, the Sidr honey was found to have broad-spectrum antibacterial activity against both Gram-positive and Gram-negative bacteria. There is a general recognition that MIC and MBC evaluation is one of the most effective and economically beneficial means of simultaneous assessment of the efficacy of multiple antimicrobials. The results of the MIC and MBC values are recorded in Table 2. Based on the obtained results, it was found that Saudi Sidr honey had a bactericidal effect against tested strains of bacteria since its MBC/MIC ratio was not greater than 2 to 3 times the MIC value of each strain.

### 3.3. Antifungal Activity of Saudi Sidr Honey

The Saudi Sidr honey showed increasing inhibitory activity with increasing honey concentrations against certain tested stains of fungi. Candida krusei and Candida albicans possessed the highest MIC levels at 1000 and 700 mg/mL, respectively. However, Candida neoformans and Candida auris showed the lowest MIC towards Sidr honey concentration of 500 mg/mL (Table 3).

### 3.4. Antibiofilm Potential of Saudi Sidr Honey

**Figure 2:**
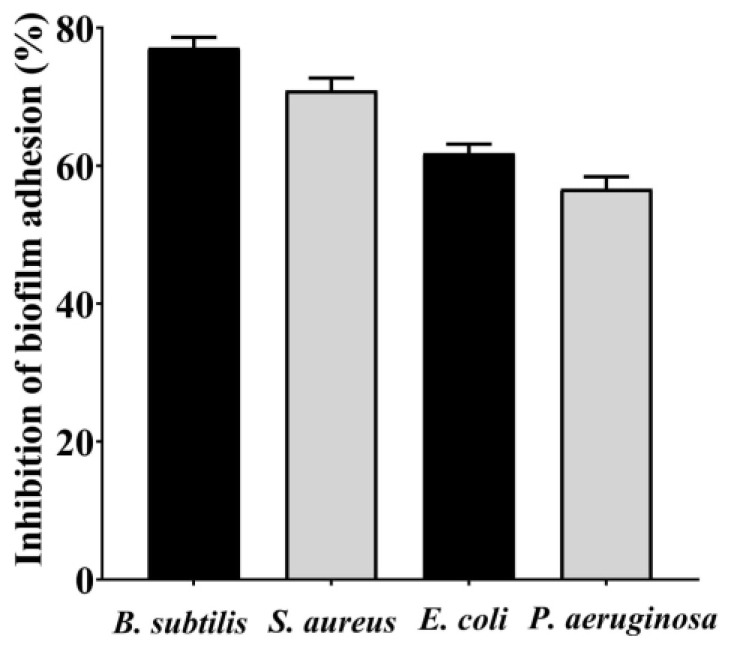
The effect of Saudi Sidr honey on the adherence ability of different pathogenic bacteria. Results were detected by a UV-Vis spectrophotometer and are presented as percentage (%) of inhibition (with respect to untreated control). Values are monitored as the mean ± SD of three independent experiments.

The antibiofilm ability of Saudi Sidr honey was determined by assessing its ability to affect the adhesion of bacterial strains to the surface. The Saudi Sidr honey was found to be capable of inhibiting the adhesion of tested bacteria to surfaces at the 1/2 MIC level. At this concentration, the adhesion ability was reduced by 77.11%, 70.88%, 61.79%, and 56.64% for *B. subtilis*, *S. aureus*, *E. coli*, and *P. aeruginosa*, respectively, at this concentration (Figure 2).

### 3.5. Saudi Sidr Honey Disrupts the Architecture of Biofilm

**Figure 3:**
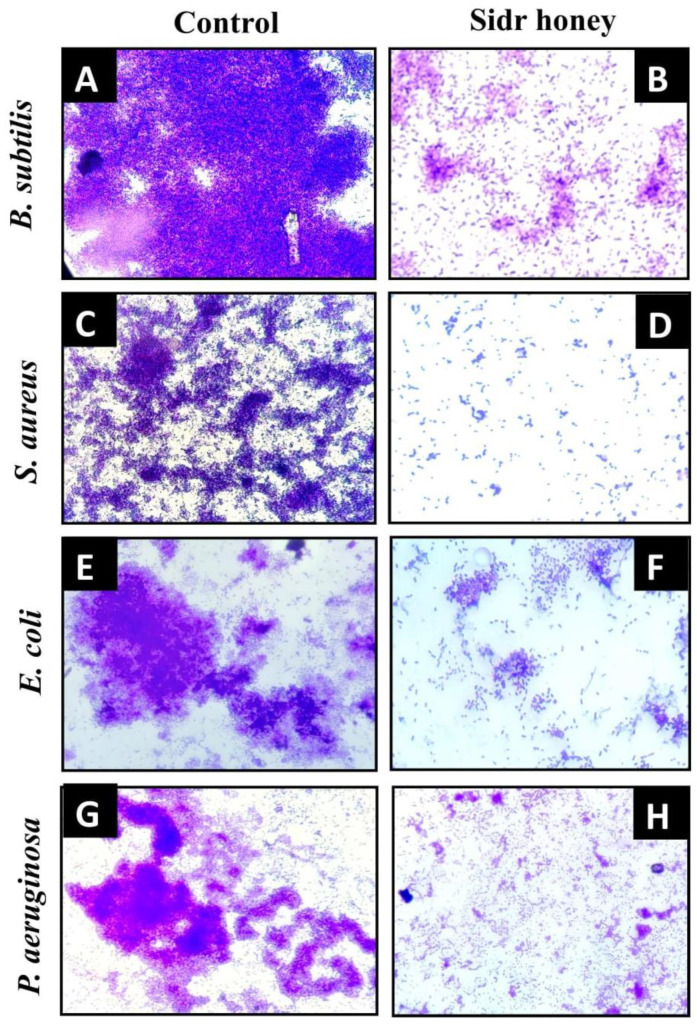
Light microscopic images of disrupted matured biofilms of tested strains formed on glass surfaces by Saudi Sidr honey at their respective sub-MIC (at 40× magnification). (**A**,**C**,**E**,**G**). Growth control; (**B**,**D**,**F**,**H**). Saudi Sidr honey treatment.

The topology of the biofilm formed by bacteria as well as the effect of Saudi Sidr honey on it was examined by light microscopy. It was found that controls showed a well-grown biofilm along with adhering bacterial cells (normal biofilms developed by bacteria), whereas treated samples revealed dispersed bacterial cells (Figure 3A–H).

### 3.6. Anti-QS Potential of Saudi Sidr Honey

**Figure 4:**
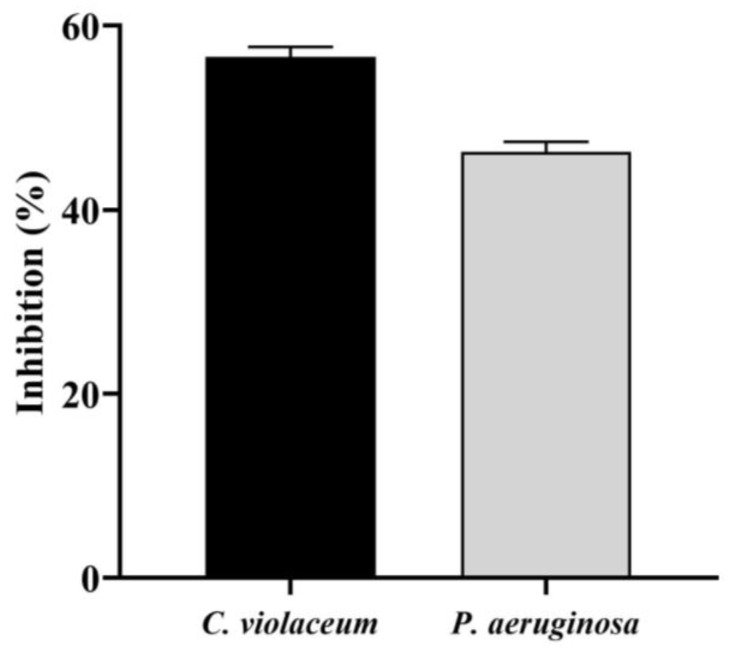
Inhibition of violacein and pyocyanin production by Saudi Sidr honey at sub-MIC concentrations. The results were detected by a UV-Vis spectrophotometer and are presented as percentage inhibition (with respect to untreated control). Values are represented as mean ± SD of three independent experiments.

The quorum sensing modulatory properties of Saudi Sidr honey were evaluated in vitro against *C. violaceum* and *P. aeruginosa*. A significant decline in pigment production was observed in both bacteria after treatment with the Sidr honey, which suggests that there was a reduction in the growth of both bacteria, as well as the presence of an anti-QS effect due to the strong inhibitory effects of the honey. The Saudi Sidr honey had MIC values of 50 mg/mL against *C. violaceum* and 70 mg/mL against *P. aeruginosa*. There was a decrease in the production of violacein and pyocyanin pigment in *C. violaceum* and *P. aeruginosa* as a result of the Sidr honey interfering with the enzyme QS activity. With a 1/2 MIC of Saudi Sidr honey, the production of violacein by *C. violaceum* was reduced to 56.63% while the production of pyocyanin by *P. aeruginosa* was reduced to 46.27% (Figure 4).

### 3.7. Antioxidant Potential of Saudi Sidr Honey

As the Saudi Sidr honey contains a high content of bioactive compounds that have previously been shown to have antioxidant properties, further exploration of its antioxidant potential in a cell-free environment was undertaken. The antioxidant potential of Saudi Sidr honey was tested for its ability to inhibit DPPH and ABTS cationic radicals, and *β*-carotene bleaching. According to the results, the Saudi Sidr honey showed antioxidant activity in a concentration-dependent manner. The highest antioxidant activities were obtained against ABTS (IC_50_—5.41 ± 0.04 mg/mL) compared to DPPH (IC_50_—7.70 ± 0.06 mg/mL) and *β*-carotene (IC_50_—≥20 mg/mL) (Table 4).

### 3.8. ADMET Properties

The ADMET properties along with the PAINS patterns of all the selected compounds were predicted using the SwissADME, ADMETLAB 2.0, and Protox-II web servers. The ADMET properties of all the compounds are reported in Table 5 and Table 6. The results showed that out of eight, five compounds (1-cyclohexylimidazolidin-2-one, 3-Butyl-3-methylcyclohexanone, 4-Butyl-3-methoxy-2-cyclo penten-1-one, 2,2,3,3-Tetramethyl cyclopropane carboxylic acid, 3,5-dihydroxy-2-(3-methylbut-2-en-1-yl)) have good ADMET properties following the Lipinski rule of five with BBB permeability and no PAINS patterns. In contrast, three compounds (glyceraldehyde, butanedioic acid, Tetrahydro-4H-pyran-4-ol) which do not have blood brain barrier (BBB) permeability were eliminated from further analysis. Examining the AMDET properties indicated that these five compounds might potentially be potent and safe candidates for further studies.

### 3.9. Molecular Docking Analysis

**Figure 5:**
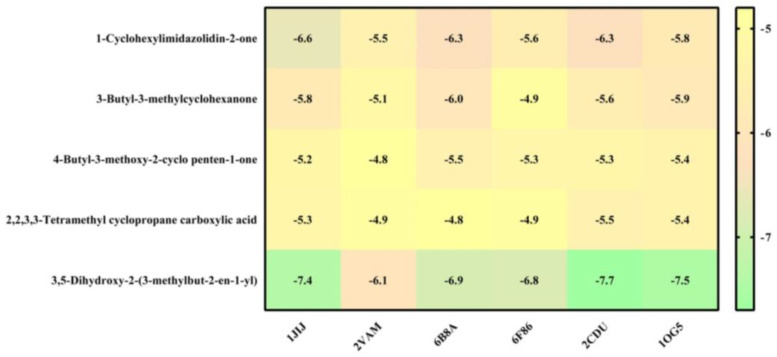
Binding affinities of top-rated poses of the ligand–receptor complex.

**Figure 6:**
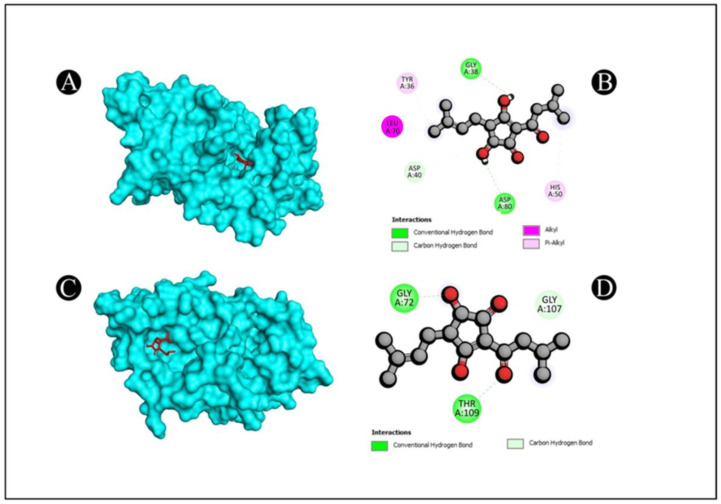
Visualization of docking analysis of 1JIJ and 3,5-dihydroxy-2-(3-methylbut-2-en-1-yl) (**A**,**B**); visualization of docking analysis of 2VAM and 3,5-dihydroxy-2-(3-methylbut-2-en-1-yl) (**C**,**D**). In A and C, ligand is presented in red color, whereas protein is presented in cyan color.

**Figure 7:**
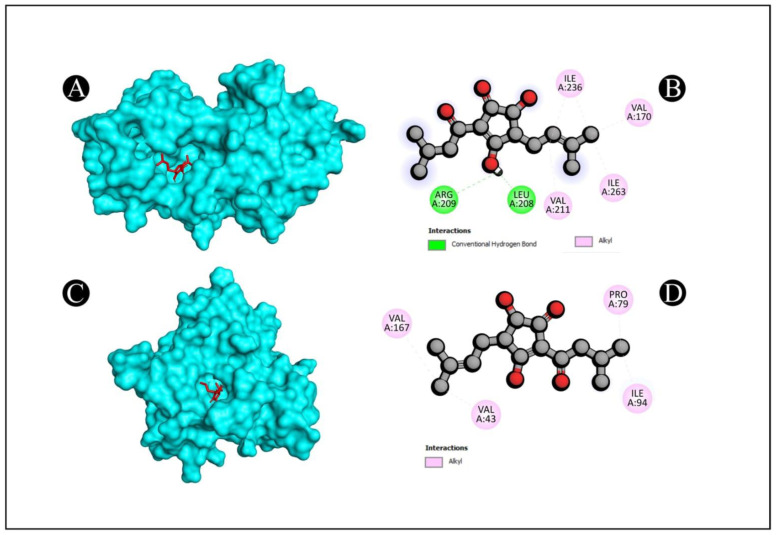
Visualization of docking analysis of 6B8A and 3,5-dihydroxy-2-(3-methylbut-2-en-1-yl) (**A**,**B**); visualization of docking analysis of 6F86 and 3,5-dihydroxy-2-(3-methylbut-2-en-1-yl) (**C**,**D**). In A and C, ligand is presented in red color, whereas protein is presented in cyan color.

**Figure 8:**
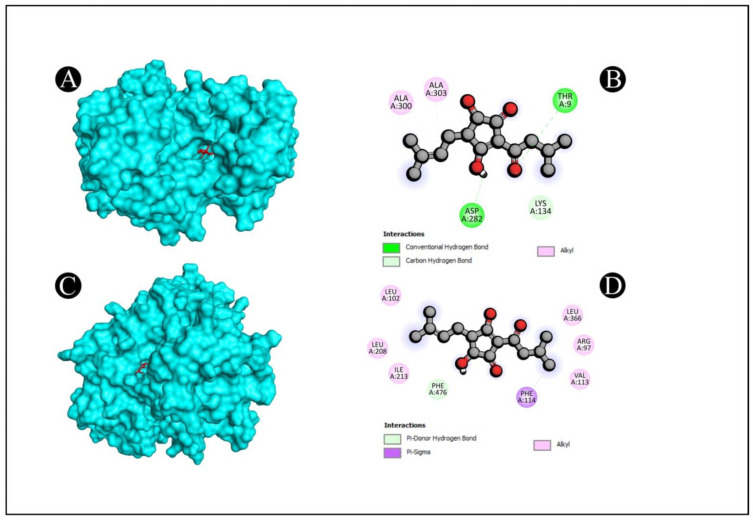
Visualization of docking analysis of 2CDU and 3,5-dihydroxy-2-(3-methylbut-2-en-1-yl), (**A**,**B**); visualization of docking analysis of 1OG5 and 3,5-dihydroxy-2-(3-methylbut-2-en-1-yl) (**C**,**D**). In A and C, ligand is presented in red color, whereas protein is presented in cyan color.

Molecular docking of five compounds, i.e., 1-cyclohexylimidazolidin-2-one, 3-butyl-3-methylcyclohexanone, 4-butyl-3-methoxy-2-cyclo penten-1-one, 2,2,3,3-tetramethyl cyclopropane carboxylic acid, and 3,5-dihydroxy-2-(3-methylbut-2-en-1-yl), with antibacterial and antioxidant potential target proteins was performed. Binding energies were calculated of the ligands against the protein targets and are reported in Figure 5. From the docking analysis, 3,5-dihydroxy-2-(3-methylbut-2-en-1-yl) showed the highest docking score with all of the six target proteins. The binding energy targets the proteins of 1JIJ, 2VAM, 6B8A, 6F86, 2CDU, and 1OG5 with docking scores of −7.4, −6.1, −6.9, −6.8, −7.7, and −7.5 kcal mol^−1^. The compounds which show higher binding affinity towards the respective proteins were selected for interaction analysis. From the interaction analysis, it was found that 3,5-dihydroxy-2-(3-methylbut-2-en-1-yl) interacts with multiple residues of target proteins, respectively. The detailed binding patterns of 3,5-dihydroxy-2-(3-methylbut-2-en-1-yl) with their respective proteins are documented in Figure 6, Figure 7 and Figure 8. There were several interactions between ligand and protein, as the ligand fitted within the binding pocket of respective proteins. The interaction between 3,5-dihydroxy-2-(3-methylbut-2-en-1-yl) and 1JIJ protein is stabilized by an interaction of two conventional hydrogen bonds (GLY38, ASP80), one carbon hydrogen bond interaction (GLY107), one alkyl interaction (LEU70), and two pi alkyl interactions (TYR36, HIS50); the 2VAM protein is stabilized by two conventional hydrogen bond interactions (GLY72, THR109) and one carbon hydrogen bond (GLY107); the 6B8A protein is stabilized by two conventional hydrogen bond interactions (LEU208, ARG209) and five alkyl interactions (VAL211, ILE236, VAL170, ILE236, ILE263); the 6F86 protein is stabilized by four alkyl protein interactions (PRO79, ILE94, VAL43, VAL167); the 2CDU protein is stabilized by two conventional hydrogen bond interactions (THR9, ASP282), two carbon hydrogen bonds (THR9, LYS134), and 2 alkyl bond interactions (ALA300, ALA303); and the 1OG5 protein is stabilized by one pi donor hydrogen bond (PHE476), one pi sigma bond interaction (PHE114), and six alkyl bond interactions (ARG97, VAL113, LEU366, LEU102, LEU208, ILE123) (Table 7).

## 4. Discussion

Antibiotic resistance is a major global public health challenge and is a growing problem at the present time [49]. Antibiotic resistance occurs when bacteria evolve to become resistant to the antibiotics that are used to treat infections, making it harder to treat bacterial infections and increasing the risk of complications. The problem of antibiotic resistance arises from the overuse and misuse of antibiotics [50]. When antibiotics are overused or used improperly, bacteria can adapt and become resistant to the drugs. This can happen through several mechanisms, including genetic mutations or the acquisition of resistance genes from other bacteria. The overuse of antibiotics is a significant problem in both human and animal health [51]. Antibiotics are often prescribed unnecessarily for viral infections or used in animal feed to promote growth and prevent disease. This overuse contributes to the development of antibiotic-resistant bacteria, which can then be transmitted between humans and animals. Addressing the problem of antibiotic resistance requires a multi-faceted approach, including reducing the overuse and misuse of antibiotics, and promoting the development of new antibiotics and alternative treatments from natural resources [52,53].

Honey has been used for medicinal purposes for thousands of years, and its antimicrobial properties have been recognized since ancient times. It contains a variety of compounds that give it its unique antimicrobial properties [54]. It has a low pH, high sugar content, and low water content, all of which contribute to its antimicrobial properties. The low pH of honey inhibits the growth of many types of bacteria, and the high sugar content creates an environment that is not conducive to bacterial growth. In addition, the low water content of honey inhibits the growth of bacteria, as most bacteria require water to grow and reproduce [55]. Honey also contains various bioactive compounds, including hydrogen peroxide, phenolic acids, flavonoids, and enzymes, which contribute to its antimicrobial properties [56]. Hydrogen peroxide is produced by the enzyme glucose oxidase, which is present in honey. This enzyme reacts with glucose to produce hydrogen peroxide, which has antimicrobial properties [57]. Phenolic acids and flavonoids are also present in honey and have been shown to have antibacterial activity [58]. Studies have shown that honey can inhibit the growth of a wide range of microorganisms, including bacteria, fungi, and viruses. Honey has been shown to be effective against antibiotic-resistant bacteria, such as methicillin-resistant *Staphylococcus aureus* (MRSA) and vancomycin-resistant *Enterococcus* (VRE) [59,60,61]. The antimicrobial properties of honey have led to its use in wound care. Honey has been shown to be effective in promoting wound healing and reducing the risk of infection. The antimicrobial properties of honey may also help to reduce inflammation and promote tissue regeneration [59]. In the present study, the Saudi Sidr honey also showed its antimicrobial activity against a variety of bacterial and fungal pathogens.

Gas chromatography-mass spectrometry (GC-MS) is a powerful analytical technique used to identify and quantify the chemical components of complex mixtures such as honey. Using GC-MS analysis, researchers have identified a wide range of compounds in honey, including sugars, organic acids, amino acids, phenolic compounds, and volatile organic compounds (VOCs) [62,63]. In the present study, different types of compounds were identified from the Saudi Sidr honey, including glyceraldehyde, butanedioic acid, Tetrahydro-4H-pyran-4-ol, 3-Butyl-3-methylcyclohexanone, 4-Butyl-3-methoxy-2-cyclopenten-1-one, 2,2,3,3-Tetramethylcyclopropanecarboxylic acid, and 3,5-dihydroxy-2-(3-methylbut-2-en-1-yl). Among the identified compounds, glyceraldehyde is a simple sugar that has been identified in honey. It is formed through the breakdown of fructose in honey and contributes to its sweetness and nutritional content. It also has potential therapeutic properties, including antioxidant and anti-inflammatory effects [64]. Butanedioic acid is a dicarboxylic acid that has been identified in honey. Its presence contributes to the sour taste and acidic pH of honey, and it has potential health benefits due to its antioxidant and anti-inflammatory properties. In addition, butanedioic acid has numerous applications in the food and pharmaceutical industries [65]. Tetrahydro-4H-pyran-4-ol is a volatile organic compound (VOC) that contributes to the aroma and flavor of honey. It is formed by the breakdown of certain sugars and other compounds in honey during the heating and storage processes. The presence of tetrahydro-4H-pyran-4-ol in honey has been linked to the floral source of the honey, with some studies suggesting that it may be more prevalent in honey derived from certain plant species. It has been found to have antioxidant and antimicrobial properties [66]. 4-Butyl-3-methoxy-2-cyclopenten-1-one is a naturally occurring organic compound that has been identified in honey. It has a number of potential health benefits and may contribute to the flavor and aroma of honey. It is a member of a class of compounds known as cyclopentenones, which are commonly found in plants and have been shown to have various biological activities [67]. The presence of 4-Butyl-3-methoxy-2-cyclopenten-1-one in honey is likely due to the fact that honeybees collect nectar and pollen from a variety of plants that contain this compound. It has been identified in several different types of honey, including thyme honey and heather honey. The compound has a number of potential health benefits, such as anti-inflammatory and antioxidant properties, and may have a role in the prevention and treatment of a variety of diseases, including cancer, cardiovascular disease, and neurodegenerative diseases. It has been described as having a sweet, woody, and floral aroma [68]. 2,2,3,3-Tetramethylcyclopropanecarboxylic acid is a naturally occurring organic acid that has been identified in honey. It has a number of potential health benefits and may contribute to the flavor and aroma of honey [69]. 3,5-Dihydroxy-2-(3-methylbut-2-en-1-yl) is a compound that can be found in honey. It is a type of flavonoid, which is a class of natural compound with antioxidant properties. This compound is also known as pinobanksin-5-methyl ether or pinobanksin-5-methyl-ether, and it has been found to have various health benefits, including anti-inflammatory and anticancer properties [70].

Antioxidants are compounds that protect the cells of the body from damage caused by free radicals, which are unstable molecules that can harm cells and contribute to the development of various diseases [71]. Honey has been reported to contain various antioxidants, including flavonoids, organic acids, phenolic acids, and enzymes such as catalase and glucose oxidase. These antioxidants are thought to help protect the body from oxidative stress and inflammation, which are involved in the development of various chronic diseases, including cancer, cardiovascular disease, and neurodegenerative diseases [72]. Several studies have investigated the antioxidant potential of honey. For example, one study found that the antioxidant activity of honey was comparable to that of vitamin C and was more effective than that of vitamin E in some cases [73]. Consuming honey with high antioxidant activity reduced oxidative stress in the body and improved lipid profiles in people with diabetes [74]. Three different methodologies, namely, DPPH, ABTS, and *β*-carotene bleaching assay, were used in this study to assess the ability of Sidr honey to scavenge respective free radicals. These values are in accordance with other antioxidant studies [75,76,77].

Moreover, biofilm formation of pathogenic bacteria is a major problem at the present time in almost all sectors, as bacteria in biofilms are often resistant to antibiotics, making them difficult to treat and leading to chronic infections [78]. Therefore, finding alternative treatments for biofilms is of great interest. Honey has been shown to have antibacterial properties, and recent studies have investigated its potential antibiofilm activity [29]. Antibiofilm activity refers to the ability of a substance to disrupt or prevent the formation of biofilms. In the present study, the antibiofilm potential of Saudi Sidr honey was determined to inhibit the adhesion ability of pathogenic bacteria. The obtained results indicated that at sub-MIC concentration, Saudi Sidr honey was capable of inhibiting the adhesion and biofilm formation in all of the tested bacteria. To further confirm that Sidr honey affects biofilm formation, the extent of microbial biofilm formation on cover slip surfaces was compared. Thus, it seems that Saudi Sidr honey exhibits significant antibiofilm activity when it is administered at sub-MIC concentrations. The antibiofilm activity of honey is thought to be due to its high sugar content [79], which can disrupt bacterial cell membranes and inhibit bacterial attachment and growth. Additionally, honey contains various enzymes and other compounds that can help break down the matrix surrounding biofilms and make them more vulnerable to treatment [80,81]. Furthermore, quorum sensing is a communication system used by bacteria to coordinate their behavior in response to population density. It plays a critical role in bacterial virulence, biofilm formation, and antibiotic resistance. Therefore, inhibiting quorum sensing is a potential strategy for treating bacterial infections [82]. The present study thus also examined the anti-QS properties of Saudi Sidr honey against *P. aeruginosa* and *C. violaceum*. The obtained results revealed that Saudi Sidr honey can effectively inhibit the QS system of both bacteria at sub-MIC concentrations via inhibiting the pyocyanin and violacein and production. Therefore, the antibiofilm activity of honey has potential applications in various fields, including medicine, food production, and environmental management.

Numerous honey samples have demonstrated positive antifungal activity in prior research, making them potential sources of antifungal agents that may be utilized in treating a variety of fungi, and particularly opportunistic fungi that cause fungus infections in immunosuppressed individuals [83]. In this investigation, the Saudi Sidr honey was shown to have additional antibacterial properties against a few fungus species from clinical and/or typical laboratory strains. The most sensitive fungal isolates examined were the clinical *C. auris* and *C. neoformans* isolates, followed by the *C. albicans* CCUG standard isolate. Because of its high resistance and colonization, the *C. auris* outbreak has led to an increase in intensive care units in hospitals. In 2021, Groot et al. demonstrated that, in contrast to other *Candida* species (*C. albicans* and *C. glabrata*), *C. auris* was the most suppressed by medical-grade honey formulations, suggesting honey antifungal activity [84]. Studies of *C. albicans* achieved conflicting findings. According to certain research, *C. albicans* is the least susceptible to honey samples at concentrations ranging from 0.1 to 100% [85,86]. However, Khosravi et al. reported utilizing several honey samples and showed that all tested honeys could achieve full suppression of MIC ranging from 29 to 56% [87], including *C. albicans*. According to other findings, *C. albicans* exhibits varying sensitivity to various honey samples [88,89,90].

The *C. krusei* standard isolated from this investigation was the least sensitive. In media contacting samples of monofloral lavender honey, Estevinho et al. [91] demonstrated lower growth rates of *C. albicans*, *C. krusei*, and *C. neoformans*. Comparing *C. albicans* and *C. neoformans*, *C. krusei* was the least sensitive. *C. albicans* and *C. neoformans*, however, were more resilient to the synthetic honey solution. There is more proof, according to Estevinho et al. [90] and DeMera and Angert [92], that the botanical honey’s origin may have a significant impact on its antibacterial action. Additionally, physico-chemical characteristics, entomological origin, and the formation of resistant strains can have an impact. Future honey research should focus on treating, in practical ways, open wounds or skin that have been infected with multi-resistant fungi isolates such as *C. auris*.

In this study, the least sensitive isolate was the CCUG standard isolate *C. krusei*. In 2011, Estevinho et al. [90] showed reduced growth rates of *C. albicans*, *C. krusei*, and *C. neoformans* in media contacting monofloral lavender honey samples. The least sensitive was *C. krusei*, and the outcome for *C. neoformans* compared to that for *C. albicans*. However, when compared to the synthetic honey solution, *C. albicans* and *C. neoformans* were more resistant. More evidence, such as that of Estevinho et al. and DeMera and Angert (2004), indicates that the botanical honey origin may play an important role in influencing the antimicrobial activity. Other factors, such as emergence of resistant strains, physico-chemical properties, and entomological origin, also have an influence. Future honey research should be directed towards practical treatment of open wounds or skin that has been colonized by multi-resistant fungal isolates, such as *C. auris* in a clinical environment.

Additionally, ADMET and molecular docking studies provide valuable information in early stages of drug discovery and development. They allow researchers to identify promising drug candidates that have desirable pharmacokinetic and pharmacodynamic properties and have the potential to bind effectively to their target proteins. This can significantly reduce the time and cost associated with drug discovery and development, as well as improve the safety and efficacy of drugs that eventually make it to market [93,94,95]. Hence, additional ADMET and molecular docking analysis of the identified bioactive compounds was carried out with the antibacterial and antioxidant target proteins. Tyrosyl-tRNA synthetase (TyrRS) is an enzyme involved in protein synthesis that attaches the amino acid tyrosine to its corresponding transfer RNA (tRNA). This enzyme has been identified as a potential drug target due to its essential role in the translation of genetic information into proteins, and its unique structural and functional properties [96]. FtsZ (Filamenting temperature-sensitive mutant Z) is a bacterial protein involved in cell division and has been identified as a potential drug target due to its essential role in bacterial cell division and its unique structural and functional properties [97]. The MvfR (Multiple Virulence Factor Regulator) is a transcription factor found in the pathogenic bacteria *P. aeruginosa* and has been identified as a potential drug target due to its role in regulating the expression of virulence factors and biofilm formation [98]. GyraseB is a bacterial enzyme involved in DNA replication and has been identified as a potential drug target due to its essential role in bacterial cell division and its unique structural and functional properties. Gyrase B is an attractive target for the development of new antibiotics, especially in the era of antibiotic resistance [99]. NAD(P)H oxidase is an enzyme that generates reactive oxygen species (ROS) in various cell types. Regarding the role of NOX in disease pathogenesis, it has emerged as a potential antioxidative drug target [100]. Cytochrome P450 (CYP) generates reactive oxygen species (ROS) as a by-product of their activity. These ROS can contribute to oxidative stress and damage cellular components such as proteins, lipids, and DNA. Given the potential role of CYP enzymes in generating ROS, there has been interest in targeting them for antioxidant activity. The obtained results in the present study indicated that the highest binding affinity of 3,5-dihydroxy-2-(3-methylbut-2-en-1-yl) was found against all of the target proteins.

Numerous prior studies have investigated the antimicrobial and antioxidant properties of Sidr honey, and their findings are consistent with our results. A previous study reported the antibacterial activity of Isis and Yemeni Sidr honey against pathogenic bacterial strains [101]. The study found that both honeys had a growth inhibitory effect on all tested bacteria, with Yemeni Sidr honey being more potently antimicrobial than the Isis honey. Sidr honey produced by dwarf honey bees was found to be a good source of antioxidants and vitamins and showed positive results against bacteria [102]. Another previous study investigated the anticancer, antimicrobial, and immunomodulatory properties, and silver nanoparticle production, of Sidr honey from three different sources. The study found that Sidr honey has antimicrobial activities and anticancer activity against HepG2 but not Hela cells [103]. A study collected Sidr honey from different geographical origins and found that the antimicrobial activity levels varied according to the origin of the honey [20]. The comparative study of Sidr honeys revealed a strong correlation between total polyphenol and flavonoid contents and significant radical scavenging activities, particularly in Egyptian and Saudi Arabian honey. The phytochemical profiling of Sidr honey from the Hail region of Saudi Arabia revealed 10 compounds belonging to several familial classes and one tripeptide. They also reported a strong antioxidant activity against DPPH (IC50 0.670 mg/mL), ABTS (IC50 3.554 mg/mL), and β-carotene (IC50 > 5 mg/mL) [104]. Sidr honey has shown a notable cytotoxic effect in a dose-dependent manner against three cancer cell lines including lung (A549), breast (MCF-7), and colon (HCT-116), with respective IC50 values of 5.203%, 6.02%, and 7.257% [105]. A study also reported the presence of bioactive compounds such as phenolic compounds, vitamin C, total phenolic contents (TPCs), radical scavenging activity (RSA), and sugars of five honey samples (Talh, Athel, Sidr, Spring flower, and Langnese) from the Hail region (Saudi Arabia) using HPLC-RID and DAD [106]. Among these, the Sidr honey sample had the highest vitamin C content of 2.59 mg/100 g^2^. These studies show that Sidr honey has significant antimicrobial and antioxidant activities, which vary depending on its origin and composition. Taking all the results of the present study into account, it can be pointed out that the Saudi Sidr honey contains a diverse group of bioactive compounds that are capable of exhibiting significant antibacterial, antifungal, antioxidant, antibiofilm, and anti-QS properties against different pathogenic microbes. However, further investigation using different techniques such as LCMS could identify the polyphenolic content of Sidr honey. Although in silico ADMET and molecular docking analysis could be helpful in proposing new lead compounds as antibacterial or antioxidant agents, molecular dynamic is needed to confirm the obtained results. 

## 5. Conclusions

The present study describes the chemical composition of Saudi Sidr honey and reports some of its biological properties, such as its ability to counteract the action of pathogenic microorganisms, scavenge free radicals, inhibit bacterial adhesion, and interfere with bacterial cell-to-cell communication. Results showed that Saudi Sidr honey contains several phytoconstituents and was able to inhibit the growth of bacteria and fungi on liquid media with low MIC values. Similarly, Saudi Sidr honey was able to inhibit the biofilm formation on a glass surface and reduce the production of violacein and pyocyanin in *C. violaceum* and *P. aeruginosa*, respectively. 

Among the identified compounds, 3,5-dihydroxy-2-(3-methylbut-2-en-1-yl exhibited a good ADMET profile and the highest binding score against antimicrobial and antioxidant targeted proteins. Further analyses are needed to confirm the possible application of Saudi Sidr honey in therapeutics.

## Figures and Tables

**Table 1:** Phytochemicals of Saudi Sidr honey identified by GC-MS analysis.

Compounds	Retention Time (min)	Molecular Formula	Chemical Structure	Fragmentation Graph
Glyceraldehyde	6.526	C_3_H_6_O_3_	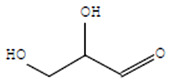	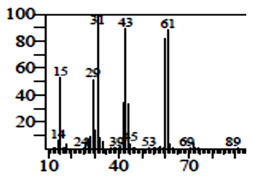
Butanedioic acid	17.681	C_5_H_8_O_4_	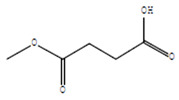	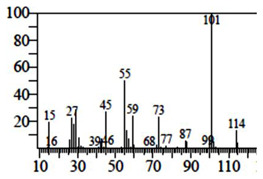
Tetrahydro-4H-pyran-4-ol	19.335	C_5_H_10_O_2_	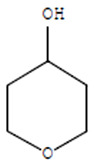	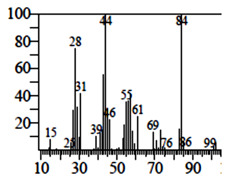
1-cyclohexylimidazolidin-2-one	23.937	C_9_H_16_N_2_O	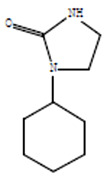	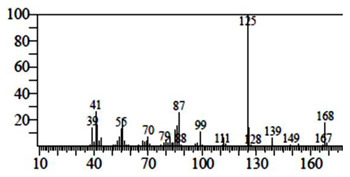
3-butyl-3-methyl-1-Cyclohexanone	25.170	C_11_H_20_O	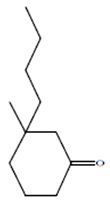	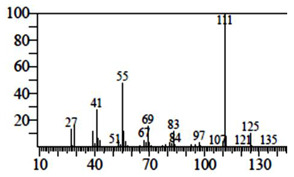
4-Butyl-3-methoxy-2-cyclopenten-1-one	25.284	C_10_H_16_O_2_	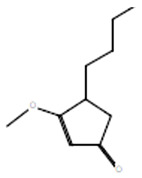	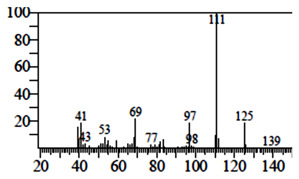
2,2,3,3-Tetramethylcyclopropanecarboxylic acid	29.099	C_13_H_24_O_2_	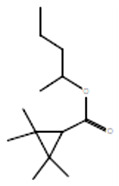	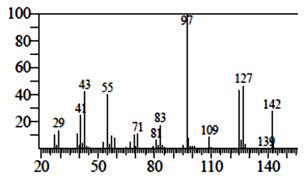
3,5-Dihydroxy-2-(3-methylbut-2-en-1-yl)	29.709	C_15_H_22_O_4_	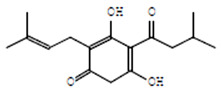	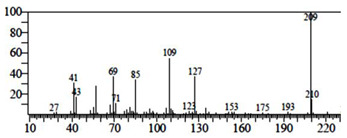

**Table 2:** Minimum Inhibitory Concentration (MIC) and Minimum Bactericidal Concentration (MBC) of Saudi Sidr honey against clinical and reference bacterial strains.

Bacterial Isolates	MIC (mg/mL)	MBC (mg/mL)	MBC/MIC Ratio
**Clinical isolates **			
*Staphylococcus aureus*	400	>450	>1.125
*Methicillin-resistant Staphylococcus aureus* 150	400	>450	>1.125
*Methicillin-resistant Staphylococcus aureus* 217	400	>450	>1.125
*Pseudomonas aeruginosa*	350	>450	>1.29
*Escherichia coli*	400	>450	>1.125
*Acinetobacter baumannii*	400	>450	>1.125
**Reference isolates**			
*Bacillus subtilis* (MTCC 121)	50	50	1
*Staphylococcus aureus* (MTCC 96)	50	100	2
*Escherichia coli* (MTCC 9537)	60	150	2.5
*Pseudomonas aeruginosa* (MTCC 741)	70	200	2.85

**Table 3:** Antifungal activity of Saudi Sidr honey.

Fungal Strains	MIC (mg/mL)
**Clinical isolates**	
*Candida auris*	500
*Cryptococcus neoformans*	500
**Standard isolates**	
*Candida krusei*	1000
*Candida albicans*	700

**Table 4:** Determination of antioxidant potential of Saudi Sidr honey.

Antioxidant Assays		IC_50_ (mg/mL)	Ascorbic Acid
Radical scavenging activity	DPPH	7.70 ± 0.06	0.02
	ABTS	5.41 ± 0.04	0.02
Bleaching assay	*β*-carotene	≥20	0.02

**Table 5:** ADME analysis of identified compounds of Saudi Sidr honey.

Sr. No.	Compound Name	MF	F (20%)	F (30%)	Caco-2	BBB	CL	C Log P_o/w_	DILI	TPSA	Bioavailability Score	Sure ChEMBL	QED	Drug-Likeness	PAINS	Lipinski
1	Glyceraldehyde	C_3_H_6_O_3_	0.006	0.004	−4.748	0.338	2.58	−1.03	0.644	57.53	0.55	0	0.435	1.174	0	Accepted
2	Butanedioic acid	C_4_H_6_O_4_	0.002	0.061	−5.903	0.254	4.976	−0.3	0.06	74.6	0.85	0	0.544	0.376	0	Accepted
3	Tetrahydro-4H-pyran-4-ol	C_5_H_10_O_2_	0.138	0.013	−4.447	0.445	9.02	0.48	0.013	29.46	0.55	0	0.472	0.64	0	Accepted
4	1-cyclohexylimidazolidin-2-one	C_9_H_16_N_2_O	0.045	0.085	−4.643	0.994	5.673	1.24	0.235	32.34	0.55	0	0.629	−0.832	0	Accepted
5	3-Butyl-3-methylcyclohexanone	C_11_H_20_O	0.227	0.903	−4.511	0.989	7.156	3.09	0.076	17.07	0.55	0	0.631	1.455	0	Accepted
6	4-Butyl-3-methoxy-2-cyclopenten-1-one	C_10_H_16_O_2_	0.981	0.978	−4.336	0.938	10.08	2.09	0.83	26.3	0.85	0	0.603	2.108	0	Accepted
7	2,2,3,3-Tetramethylcyclopropanecarboxylic acid	C_8_H_14_O_2_	0.004	0.001	−5.208	0.759	1.516	1.67	0.88	37.3	0.85	0	0.605	1.002	0	Accepted
8	3,5-Dihydroxy-2-(3-methylbut-2-en-1-yl)	C_15_H_22_O_4_	0.003	0.006	−4.473	0.082	6.646	2.19	0.944	68.28	0.85	0	0.544	0.975	1	Accepted

Abbreviations—F (20%) = 20% bioavailability, F (30%) = 30% bioavailability, BBB = blood brain barrier, CL = clearance, C Log P_o/w_ = average of lipophilicity, DILI = drug induced liver injury, TPSA = topological polar surface area, QED = quantitative estimate of drug-likeness, PAINS = pan assay interference compounds.

**Table 6:** Toxicity analysis of identified compounds of Saudi Sidr honey using Protox-II Server.

Sr. No.	Compound	Canonical Smiles	LD_50_	Hepatotoxicity	Carcinogenicity	Immunotoxicity	Mutagenicity	Cytotoxicity
1	Glyceraldehyde	C(C(C=O)O)O	200 mg/kg	Inactive	Inactive	Inactive	Inactive	Inactive
2	Butanedioic acid	C(CC(=O)O)C(=O)O	2260 mg/kg	Inactive	Inactive	Inactive	Inactive	Inactive
3	Tetrahydro-4H-pyran-4-ol	C1COCCC1O	2994 mg/kg	Inactive	Inactive	Inactive	Inactive	Inactive
4	1-Cyclohexylimidazolidin-2-one	C1CCC(CC1)N2CCNC2=O	2000 mg/kg	Inactive	Inactive	Inactive	Inactive	Inactive
5	3-Butyl-3-methylcyclohexanone	CCCCC1(CCCC(=O)C1)C	500 mg/kg	Inactive	Inactive	Inactive	Inactive	Inactive
6	4-Butyl-3-methoxy-2-cyclopenten-1-one	CCCCC1CC(=O)C=C1OC	2800 mg/kg	Inactive	Inactive	Inactive	Inactive	Inactive
7	2,2,3,3-Tetramethyl cyclo propane carboxylic acid	CC1(C(C1(C)C)C(=O)O)C	600 mg/kg	Inactive	Inactive	Inactive	Inactive	Inactive
8	3,5-Dihydroxy-2-(3-methylbut-2-en-1-yl)	CC(C)CC(=O)C1=C(C(C(C1=O)O)CC=C(C)C)O	2000 mg/kg	Inactive	Inactive	Inactive	Inactive	Inactive

**Table 7:** Interactive active site residues’ top-rated poses of biosurfactants with target proteins.

Sr. No.	Protein	Receptor-Ligand	Interaction Type	Distance
1	1JIJ	N:UNK1:H - A:GLY38:O	Conventional Hydrogen Bond	2.37128
		N:UNK1:H - A:ASP80:OD2	Conventional Hydrogen Bond	2.44926
		N:UNK1:C - A:ASP40:OD1	Carbon Hydrogen Bond	3.6421
		N:UNK1:C - A:LEU70	Alkyl	4.02983
		A:TYR36 - N:UNK1:C	Pi-Alkyl	4.98438
		A:HIS50 - N:UNK1:C	Pi-Alkyl	4.51308
2	2VAM	A:GLY72:HN - N:UNK1:O	Conventional Hydrogen Bond	1.85211
		A:THR109:HN - N:UNK1:O	Conventional Hydrogen Bond	2.48596
		A:GLY107:CA - N:UNK1:O	Carbon Hydrogen Bond	3.5012
3	6B8A	N:UNK1:H - A:LEU208:O	Conventional Hydrogen Bond	2.71778
		N:UNK1:H - A:ARG209:O	Conventional Hydrogen Bond	2.34106
		A:VAL211 - N:UNK1	Alkyl	4.30848
		A:ILE236 - N:UNK1	Alkyl	4.42091
		N:UNK1:C - A:VAL170	Alkyl	4.54508
		N:UNK1:C - A:ILE236	Alkyl	4.54213
		N:UNK1:C - A:ILE263	Alkyl	4.77393
4	6F86	N:UNK1:C - A:PRO79	Alkyl	4.45171
		N:UNK1:C - A:ILE94	Alkyl	4.92383
		N:UNK1:C - A:VAL43	Alkyl	4.6844
		N:UNK1:C - A:VAL167	Alkyl	4.34783
5	2CDU	A:THR9:HG1 - N:UNK1:O	Conventional Hydrogen Bond	2.06555
		N:UNK1:H - A:ASP282:OD1	Conventional Hydrogen Bond	2.39425
		A:THR9:CB - N:UNK1:O	Carbon Hydrogen Bond	3.53136
		A:LYS134:CE - N:UNK1:O	Carbon Hydrogen Bond	3.65143
		A:ALA300 - N:UNK1	Alkyl	4.862
		A:ALA303 - N:UNK1	Alkyl	4.57296
6	1OG5	N:UNK1:H - A:PHE476	Pi-Donor Hydrogen Bond	3.28989
		N:UNK1:C - A:PHE114	Pi-Sigma	3.70704
		N:UNK1:C - A:ARG97	Alkyl	4.85544
		N:UNK1:C - A:VAL113	Alkyl	4.59957
		N:UNK1:C - A:LEU366	Alkyl	4.60615
		N:UNK1:C - A:LEU102	Alkyl	5.00293
		N:UNK1:C - A:LEU208	Alkyl	4.63598
		N:UNK1:C - A:ILE213	Alkyl	4.69187

## Data Availability

All data that support the findings of this study are available within the article.
